# Porous titanium scaffolds modified with Zeolitic Imidazolate Framework (ZIF-8) with enhanced osteogenic activity for the prevention of implant-associated infections

**DOI:** 10.3389/fchem.2024.1452670

**Published:** 2024-08-29

**Authors:** Valentina Di Matteo, Maria Francesca Di Filippo, Barbara Ballarin, Francesca Bonvicini, Maria Rosa Iaquinta, Silvia Panzavolta, Elisa Mazzoni, Maria Cristina Cassani

**Affiliations:** ^1^ Department of Industrial Chemistry “Toso Montanari”, University of Bologna, Bologna, Italy; ^2^ Department of Chemistry “G. Ciamician”, University of Bologna, Bologna, Italy; ^3^ Department of Pharmacy and Biotechnology, University of Bologna, Bologna, Italy; ^4^ Department of Medical Sciences, Section of Experimental Medicine, University of Ferrara, Ferrara, Italy; ^5^ Department of Chemical, Pharmaceutical and Agricultural Sciences, University of Ferrara, Ferrara, Italy; ^6^ Laboratory for Technologies of Advanced Therapies (LTTA), University of Ferrara, Ferrara, Italy

**Keywords:** zeolitic imidazolate framework-8 (ZIF-8), titanium scaffolds, hydroxyapatite, gelatin-A, osteoblasts, bacterial infections, anti-adhesion properties

## Abstract

In this study, zeolitic imidazolate framework 8 (ZIF-8) was coated on porous Ti6Al4V scaffolds, either bare or previously modified using hydroxyapatite (HA) or HA and gelatin (HAgel), via a growing single-step method in aqueous media using two contact times at 6 h and 24 h. The coated scaffolds termed ZIF-8@Ti, ZIF-8@HA/Ti, and ZIF-8@HAgel/Ti were characterized via scanning electron microscopy (SEM), powder X-ray diffraction (PXRD), attenuated total reflectance-Fourier transform infrared (ATR-FTIR), and molecular plasma-atomic emission spectroscopy (MP-AES). In order to assess the cell proliferation rate, the cytocompatibility of the scaffolds was evaluated in primary osteoblasts (hOBs) using alamarBlue assay, while the osteoconductivity was analyzed in hOBs using a real-time approach, evaluating the expression of secreted phosphoprotein 1 (SPP1). Osteopontin, which is the protein encoded by this gene, represents the major non-collagenous bone protein that binds tightly to HA. The scaffolds were shown to be non-cytotoxic based on hOB proliferation at all time points of analysis (24 h and 72 h). In hOB cultures, the scaffolds induced the upregulation of SPP1 with different fold changes. Some selected scaffolds were assayed *in vitro* for their antibacterial potential against *Staphylococcus epidermidis*; the scaffolds coated with ZIF-8 crystals, regardless of the presence of HA and gelatin, strongly inhibited bacterial adhesion to the materials and reduced bacterial proliferation in the culture medium, demonstrating the suitable release of ZIF-8 in a bioactive form. These experiments suggest that the innovative scaffolds, tested herein, provide a good microenvironment for hOB adhesion, viability, and osteoconduction with effective prevention of *S. epidermidis* adhesion.

## 1 Introduction

Titanium and its alloys, especially Ti_6_Al_4_V, are widely used in the industry and in the biomedical field, particularly in bone fusion, bone fixation, and joint replacement surgery (Kaur and Singh, 2019). Due to their excellent mechanical properties, low density, good corrosion resistance, and biocompatibility, these materials have been successfully employed as artificial implants in dental and orthopedic surgery for decades.

Bone response and implant success depend on the chemical and physical properties of the biomaterial surface in direct contact to biological tissue (Garg et al., 2021; Han et al., 2023; Akay and Yaghmur, 2024). However, the bioinertness and poor antibacterial activity of titanium-based implants prevent good osseointegration of surrounding natural tissues, especially during an infection (Phillips et al., 2006; Chouirfa et al., 2019). It has been demonstrated that the integration with bone tissue can be improved and accelerated by the presence of a proper coating onto the metal surface. As calcium phosphates are bioactive and osteoconductive and promote direct attachment to bone, several calcium phosphate coatings have been extensively applied with the aim of improving fixation between hard tissue and metal implants (LeGeros, 2002). Hydroxyapatite [HA, Ca_10_(PO_4_)_6_(OH)_2_] has gained much recognition as a bone filler because of its high structural similarity to the mineral phase of bone tissue. HA has a unique ability to enhance biochemical interactions with biological systems, thus achieving the regeneration of the hosted bone toward the grafted material. Therefore, coating Ti with nanocrystalline HA has proved to enhance the formation of new bone tissue within a short time by promoting osteoblast adhesion and inducing the biomineralization of the implant (Bigi et al., 2005).

The prevention of implant-associated bone infections is still a major challenge for both orthopedic and dental surgeries (ter Boo et al., 2015). Incorporation of antibiotics within HA coatings may produce significant benefits, such as superior osteoconductivity and bactericidal properties (Abdulkareem et al., 2015; Geuli et al., 2019). Nevertheless, overuse of common antibiotics has led to the significant prosperity of antibiotic-resistant bacteria, which has become a major concern globally. Therefore, several efforts have been made to find alternative materials as effective antimicrobial agents. To this aim, metal-based nanoparticles such as silver nanoparticles and various micro- and nano-sized metal oxides, such as TiO_2_, CuO, Fe_2_O_3_, and ZnO, demonstrated a wide antibacterial spectrum, high stability, and relative non-toxicity (Mishra et al., 2017; Shimabukuro, 2020; Raja et al., 2023).

Coupling of metal or metal oxide nanoparticles with Ti-based scaffolds endowed the materials with antibacterial properties, and several approaches are reported in the literature (Kheirmand-Parizi et al., 2024). Metal–organic frameworks (MOFs) are a class of ordered nanoporous solid crystals that have attracted growing interest for applications in the biomedical field, thanks to their large surface area, low toxicity, and high porosity (Furukawa et al., 2013; Kaskel, 2016; Wang et al., 2023). In particular, zeolitic imidazolate framework-8 (ZIF-8), constructed from 2-methylimidazole and zinc ions, is a subclass of MOFs that are chemically stable in aqueous and basic media and have surface areas up to ca. 1,800 m^2^g^−1^ (Park et al., 2006; Venna et al., 2010; Ploetz et al., 2020; Kouser et al., 2022). These physical properties coupled with its excellent thermal stability and pH-responsive dissolution behavior (in acidic solutions) have motivated the investigation of ZIF-8 for biomedical applications (Sun et al., 2020; Wang et al., 2020; Yang and Yang, 2020; Abdelhamid, 2021; Pourmadadi et al., 2023). To the best of our knowledge (Tang et al., 2024), only a few papers have reported the successful coating of bare Ti-based scaffolds by using ZIF-8. Some of these papers reported the further addition of the antibiotic levofloxacin (Tao et al., 2020), silver nitrate to enhance the antibacterial activity (Li et al., 2022), or bioactive molecules such as naringin to improve osteointegration (Wang et al., 2023).

Indeed, recently, some of the authors demonstrated the feasibility of *in situ* functionalization of natural cellulose fibers with ZIF-8 crystals using water as a unique solvent that leads to the production of an effective wound dressing antibacterial material (Di Matteo et al., 2023). In this frame, ZIF-8 was chosen as the active material for the functionalization of Ti_6_Al_4_V porous scaffolds via an *in situ* single-step method carried out at room temperature and in aqueous media (Chen et al., 2017; Zhang et al., 2017). Furthermore, with the aim to improve osteointegration, two different coatings were performed on porous Ti_6_Al_4_V before treatment with ZIF-8. Scaffolds were previously coated with nanocrystalline HA by means of using a biomimetic method (Bigi et al., 2005), and, as a further improvement, HA was deposited in the presence of gelatin, a protein obtained by thermal denaturation or physical and chemical degradation of collagen (Lien et al., 2009; Gomez-Guillen et al., 2011) that had previously demonstrated beneficial effects on osteoblast proliferation and differentiation when co-precipitated with nanocrystalline HA (Bracci, 2012).

Coating of Ti scaffolds with HA, gelatin A, and ZIF-8 reduces the risk of implant-related infections and promotes long-term cellular regeneration processes, improving the activity of osteoblasts. The antibacterial potential of the coated Ti scaffolds was assessed against the *Staphylococcus epidermidis* strain, which is a ubiquitous commensal bacterium responsible for bone implant-associated osteomyelitis (Kavanagh et al., 2018) previously reported as a susceptible pathogen to ZIF-8 crystals (Di Matteo et al., 2023).

## 2 Materials and methods

### 2.1 Materials

Chemicals were used as received from Sigma-Aldrich (now Merck KGaA, Darmstadt, Germany), if not stated otherwise; ultrapure water purified using the Milli-Q Plus system (Millipore Co., resistivity over 18 MΩ cm, Burlington, VT, United States) was used in all experiments. Porous Ti_6_Al_4_V scaffolds (hereafter simply indicated as the Ti scaffold) obtained by additive manufacturing with shape and morphology, as shown in Supplementary Figure S1 (1 × 1 × 0.2 cm, av. weight 0.32 g), were kindly provided by MT ORTHO, Catania, Italy.

### 2.2 Pretreatment of Ti substrates

Each titanium scaffold was first etched using a freshly prepared Kroll’s mixture using the following protocol (Bigi et al., 2005). In a 50-mL volumetric flask, 200 µL of HNO_3_ (65 wt%) and 100 µL of HF (40 wt%) were mixed and brought to volume with water. Immediately after that, the Ti scaffold was immersed in a beaker containing 50 mL of Kroll’s mixture and then sonicated for 10 min. After the treatment, the scaffold was repeatedly washed using distilled water and dried in air at room temperature.

### 2.3 Coating of Ti substrates with hydroxyapatite and hydroxyapatite/gelatin type A

The coating with HA was performed according to the following protocol (Bigi et al., 2005). First, the HEPES buffer solution was prepared dissolving 1.72 g of HEPES sodium salt in 100 mL of water adjusting the final pH to 7.2. Solution A: 37 mg of CaCl_2_·2H_2_O was dissolved in 50 mL of HEPES and placed in an incubator at 37°C. Solution B: 95 mg of Na_3_PO_4_·12H_2_O and 151 mg of NaHCO_3_ were dissolved in 50 mL of HEPES and placed in an incubator at 37°C. Successively, solutions A and B were mixed by stirring in a beaker containing freshly etched titanium scaffolds. Samples were kept at 37°C overnight, thoroughly washed using water, and then dried in air at room temperature. The samples were labeled HA/Ti. The coating with HAgel was obtained by following the same procedure as shown above, but 0.1% (w/V) gelatin type A (300 Bloom, Merck) was added to the HEPES buffer. The samples were labeled HAgel/Ti (Bracci, 2012).

### 2.4 Deposition of ZIF-8

The *in situ* deposition of ZIF-8 on the scaffolds was carried out by varying the Zn:2-HmIM (2-methylimidazole) molar ratio and the deposition time (Hoop et al., 2018; Velásquez-Hernández et al., 2019). In a typical procedure using the Zn:2-HmIM molar ratio of 1:16, a 0.08 M solution of Zn(OAc)_2_∙2H_2_O was prepared by dissolving 88 mg of Zn(OAc)_2_∙2H_2_O (0.40 mmol, MW: 219.51 g/mol) in 5 mL of water. Subsequently, this solution was transferred in a beaker containing a previously etched Ti scaffold and left at room temperature for approximately 30 min to allow the absorption of Zn^2+^ ions. Then, a 1.28 M solution of 2-HmIM, prepared by dissolving 0.527 g of this compound (6.42 mmol, MW: 82.20 g/mol) in 5 mL of H_2_O, was poured into the beaker containing the scaffold to initiate the formation of ZIF-8 at room temperature without stirring. After a few minutes, a milky suspension was formed, highlighting the onset of the reaction. The scaffolds were kept into this suspension for different periods of time (3, 6, and 24 h) and then immersed 10 times in water to remove all unabsorbed ZIF-8. All the scaffolds were allowed to dry in air at room temperature and then thermally activated at 100°C and 10^−2^ mbar to remove the excess of 2-HmIM by sublimation (time of treatment = 1 h). The supernatant suspensions were centrifuged (Centrifuge Remi XSR-8D, 6,000 rpm for 20 min), and after washing with water and centrifugation, the collected ZIF-8 powders were dried at 60°C for 24 h. After being thermally activated at 100°C and 10^−2^ mbar, they were stored in a desiccator for further characterizations. For the syntheses made with a Zn:2-HmIM molar ratio of 1:10, the following quantities were employed: 301 mg of Zn(OAc)_2_ 2H_2_O in 5 mL of water and 1.12 g of 2-HmIM in 5 mL of water.

### 2.5 Instrumentation

Powder X-ray diffraction (PXRD) patterns were recorded in the reflection mode by using a Philips X’Celerator diffractometer equipped with a graphite monochromator. The 2θ range was from 4° to 45° with a step size of 0.100° and a time per step of 120 s. CuKα (40 mA, 40 kV, and 1.54 Å) was used. Morphological investigation was performed using a scanning electron microscopy (SEM), and the images were obtained using a Leica/Cambridge Stereoscan 360 device with INCA software; Digimizer software (version 5.8.0, MedCalc Software Ltd., Ostend, Belgium) was used to estimate the mean dimensions, averaging the measurements over at least 100 data points per sample. The overall amount of zinc present in different samples was determined by means of Agilent 4210 molecular plasma-atomic emission spectroscopy (MP-AES) using the zinc line at 481.053 nm. The analyses were conducted by comparison with five calibration standards (2, 20, 30, 50, and 100 ppm) and prepared with dilution to 100 mL of a 1,000 ppm zinc standard (CARLO ERBA Reagents, Milan, Italy). Before performing the analyses, each scaffold was weighed and then immersed for 30 min in 10 mL of 1.0 M nitric acid (NORMATOM^®^, VWR International) at room temperature. Successively, the solution was transferred in a 25-mL volumetric flask and brought to volume using 1.0 M of nitric acid; results from these analyses represent the mean value of three different determinations. Attenuated total reflectance–Fourier transform infrared (ATR-FTIR) analyses were performed using a PerkinElmer Spectrum Two spectrophotometer, equipped with a Universal ATR accessory; all spectra were recorded as an average of 40 scans (range 4,000–400 cm^−1^ with a resolution of 0.5 cm^−1^).

### 2.6 Biological evaluation

All the scaffolds were sterilized via UV (253 nm) for 2 h before biological evaluation. The primary cell cultures employed to analyze the cytocompatibility and osteoconductivity of the scaffolds are human primary osteoblasts (hOBs) commercially available (Cat. n. LOCC2538, Lonza, Milan, Italy). On the other hand, the *in vitro* antibacterial activity of the Ti scaffolds was assayed against the reference strain of *S. epidermidis* (ATCC 12228).

### 2.7 Osteoblast proliferation

The hOBs were cultured using a specific culture medium, i.e., OGM Osteoblast Growth BulletKit (Cat. n. LOCC3207, Lonza, Milan, Italy), which is composed of osteoblast basal medium, fetal bovine serum (FBS), gentamicin–amoxicillin (G/A), and ascorbic acid. Osteoblastic cells were seeded on the biomaterials, as previously described, in 24-well plates with a density of 5.0 × 10^4^ cells/well and kept at 37°C at 5% CO_2_ until analysis (Mazzoni et al., 2021).

The proliferation of hOBs attached and grown on scaffolds was evaluated using the alamarBlue assay^TM^ (Invitrogen, Milan, Italy). Indeed, cells were incubated in medium with 5% alamarBlue reagent for 3 h at 37°C (Mazzoni et al., 2021). To generate a calibration curve, a concentration of 8.0 × 10^4^ cells and serial 1:2 dilutions were seeded, consisting of the scalar concentration of cells up to 5.0 × 10^3^ cells. In brief, cells were incubated with a solution of 10% alamarBlue in medium for 3.5 h at 37°C. The supernatant optical density was measured at a 570 nm wavelength and at a 620 nm reference wavelength using a spectrophotometer (Thermo Electron Corporation, model Multiskan EX, Helsinki, Finland) after (T1) 24 h and (T2) 72 h. All reactions were performed in triplicate.

### 2.8 Osteoblast gene expression

Total RNA was isolated using the TRIzol reagent (Thermo Fisher, Milan, Italy), according to the manufacturer’s instructions at 72 h (Mazzoni et al., 2017). Total extracted RNA was quantified by using a NanoDrop spectrophotometer (ND-1000; Wilmington, Delaware) (Martini et al., 2020). Purified RNA was reverse-transcribed to cDNA using the ImProm-II Reverse Transcriptase (Promega, Milan, Italy). cDNAs were stored at – 20°C until the time of analysis. Real-time PCR was performed for the detection of secreted phosphoprotein 1 (SPP1). SPP1, which encodes for osteopontin, was analyzed in hOBs grown on scaffolds using the TaqMan™ Gene Expression Assay (FAM) (Hs00959010-OPN-FAM, Life Technology, Milan, Italy) and thermocycler CFX96 (Bio-Rad, Milan, Italy). For data analysis, fold changes of the gene expression were calculated by using the 2^−ΔΔCT^ method, whereas housekeeping genes that were employed as control were used to normalize results. The gene expression level of the target gene was calculated by normalization to the reference gene RLP13A (HS 01578912-RLP13A-VIC, Life Technology, Milan, Italy).

### 2.9 Antibacterial studies

Bacteria were routinely grown on a 5% blood agar plate (Biolife Italiana s.r.l., Milan, Italy) at 37°C, and 24-h cultures were used for the experiments. For the anti-adhesion assays, bacterial suspensions were prepared in Tryptic soy broth (TSB) with 2% glucose and adjusted at 0.25 OD_600nm_ (optical density); thereafter, 1 mL of suspension was transferred onto a 24-well plate, and the Ti scaffolds were inserted. After 4 h of incubation at 37°C with gentle shaking, OD_600nm_ values of the bacterial cultures were measured to determine *S. epidermidis* growth, and the Ti scaffolds were harvested and carefully washed in PBS buffer (0.1 M; pH 7.2) to remove non-adhered cells. Subsequently, the scaffolds were transferred into a new 24-well plate and incubated in 1 mL of fresh medium using 10% alamarBlue^TM^ HS reagent (Invitrogen, Milan, Italy) for 2 h at 37°C. Finally, aliquots of the supernatants were transferred to a 96-well plate, and the OD_550/630nm_ value was read with a microplate reader to assess the metabolic activity of the adhered *S. epidermidis*.

Growth curves of *S. epidermidis* were also determined by monitoring the turbidity of the bacterial cells for up to 24 h of incubation at 37°C in dynamic conditions (300 rpm). In detail, an overnight culture was prepared in MH (Mueller–Hinton) broth; thereafter, 1 mL of the bacterial suspension diluted 1:100 in the same medium was transferred into a 24-well plate, in which the Ti scaffolds were inserted. Growth rates were determined by measuring the OD_600nm_ value at defined intervals.

For the anti-biofilm assays, bacterial suspensions were prepared at 0.08 OD_600nm_, 10-fold diluted in TSB, and incubated for 90 min with the Ti scaffolds, as previously described. Subsequently, Ti scaffolds were washed in PBS and further incubated in fresh medium at 37°C, under static conditions for 48 h, allowing for biofilm formation. The Ti scaffolds were removed from the cultures to quantify the biomass of the biofilm layering the samples through crystal violet (CV) staining. In brief, Ti scaffolds were transferred into a new 24-well plate, washed in PBS buffer, dried at 60°C for 1 h, and then incubated for 30 min with 1 mL of a CV solution (0.1% in water). Then, the scaffolds were washed twice with water to remove the unbound dye, and CV was dissolved using 1 mL of 95% ethanol for 30 min. Finally, aliquots of colored supernatants were transferred to a 96-well plate, and OD_550nm_ was read. The percentage inhibition of biofilm formation in different experimental conditions was calculated as relative to the biofilm mass formed in the plain Ti scaffolds.

### 2.10 Zn^2+^ ion release kinetics

To analyze the release of Zn^2+^ ions, the ZIF-8 functionalized Ti scaffolds were placed in a 24-well plate and incubated with 1 mL of MH broth at 37°C under constant and slow oscillation (300 rpm). At predetermined time intervals, the solutions were harvested and renewed with the same amount of fresh medium. This procedure was selected to guarantee dynamism in the elution environment, mimicking the exchange of physiological fluid. The harvested solutions were diluted in volumetric flasks up to a volume of 10 mL by using water, and the Zn^2+^ content was evaluated by means of a MP-AES using the zinc line at 481.053 nm. Data were expressed as Zn^2+^ percentage values, referring to the total amount of released zinc ions.

### 2.11 Scanning electron microscopy of bacterial cells on Ti scaffolds

Ti scaffolds recovered at the end of anti-adhesion assay were observed through SEM analysis to observe *S. epidermidis* cells. For this purpose, the Ti scaffolds were harvested, washed twice with cold PBS, and fixed in PBS using 2.5% glutaraldehyde for 2 h at 4°C. After fixation, scaffolds were rinsed with PBS buffer and dehydrated by gradual dehydration in ethanol (30%, 70%, and 96% in v/v) at room temperature. Dehydrated samples were sputter-coated with gold (30 mA, t = 2.5 min) prior to the observation.

### 2.12 Statistical analysis

Statistical analyses were carried out using Prism 8 software (GraphPad 8.0, San Diego, CA, United States). Data obtained from alamarBlue-based assays, both cellular and bacterial determinations, were analyzed with the two-way ANOVA and Tukey’s test. For gene expression analysis, ΔCT values were calculated, and one-way ANOVA with Tukey’s test was used. Gene expression was calculated as a fold change (FC) relative to Ti-based materials, considered the control group, using the 2^−ΔΔCT^ method. Ribosomal protein L13a (RPL13A) was employed as a housekeeping gene to normalize data. The *in vitro* experiments were performed in triplicate. A value of *p* < 0.05 was considered significant.

## 3 Results and discussion

### 3.1 Preparation and characterization of etched Ti scaffolds

In order to evaluate the optimal Zn:2-HmIM molar ratio and deposition time, preliminary *in situ* deposition of ZIF-8 was carried out on freshly etched Ti-based scaffolds. The resulting phase deposition and morphology were assessed and evaluated by means of PXRD and SEM. A total of six samples represented in Table 1 were prepared with a Ti-etched scaffold, which is used as a reference.

**TABLE 1:** Preparation of ZIF-8@Ti samples^a^.

Batch	Sample name (h)	Zn (OAc)_2_∙2H_2_O (mM)^b^	2-HmIM (mM)^b^	Molar ratio Zn:2-HmIM	Deposition time (h)
1	ZIF-8@Ti-1_3	80	1,280	1:16	3
	ZIF-8@Ti-1_6				6
	ZIF-8@Ti-1_24				24
2	ZIF-8@Ti-2_3	274	2,740	1:10	3
	ZIF-8@Ti-2_6				6
	ZIF-8@Ti-2_24				24

^a^ All samples were prepared in triplicate.

^b^ Concentrations of the premix solution.

**FIGURE 1:**
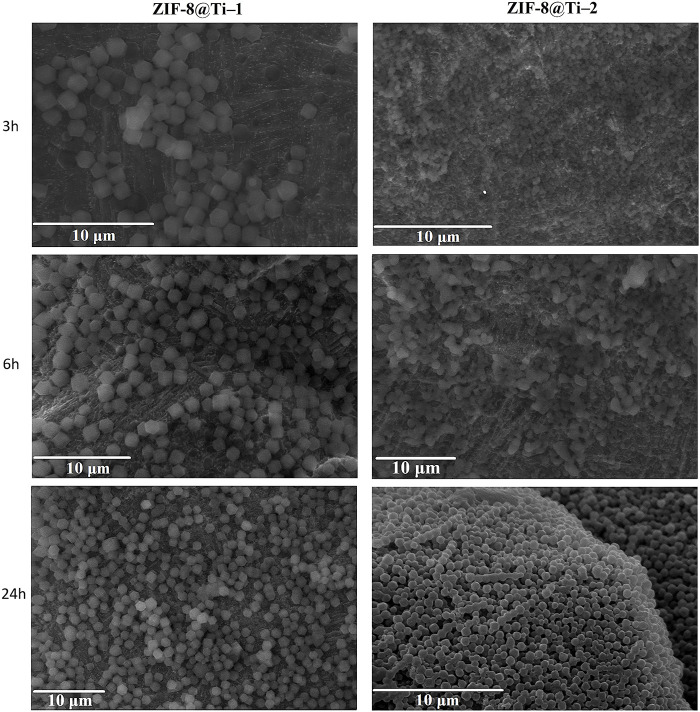
SEM images obtained on the samples listed in Table 1 (scale bar = 10 µm).

The SEM images, as shown in Figure 1, confirmed the presence of ZIF-8 crystals on all the treated Ti-based scaffolds. However, according to Jian et al. (2015), crystals with homogeneous dimensions and a well-defined morphology for each contact time (samples termed ZIF8@Ti-1) are obtained when a higher Zn:2-HmIM molar ratio (1:16) was used. In fact, in this case (see Figure 1), the characteristic rhombic dodecahedron ZIF-8 crystals are well-visible already after 3 h, and their presence proportionally increases in number with time; after 6 h, the scaffolds are homogeneously and completely covered. The average size of ZIF-8 crystals on the ZIF-8@Ti-1 scaffold after 3, 6, and 24 h is the same and found to be (1.1 ± 0.2) µm. On the contrary, with a ratio of 1:10 (samples termed ZIF-8@Ti-2), a homogeneous and well-defined crystalline morphology is obtained only after 24 h with an average size of (1.2 ± 0.2) µm. SEM images taken on the coated scaffolds ZIF-8@Ti stored in a desiccator for 3 months showed no changes in size and morphology during this time frame, proving their good stability over time.

**FIGURE 2:**
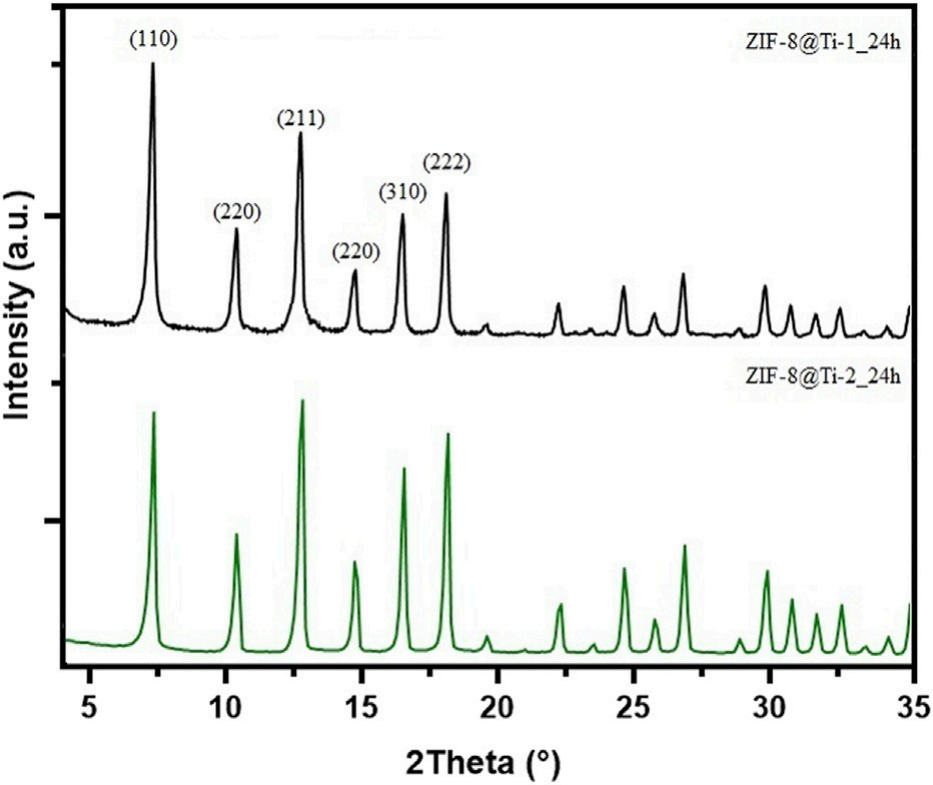
Comparison between PXRD patterns obtained from ZIF-8@Ti-1_24h (black) and ZIF-8@Ti-2_24h (green).

To confirm the formation of *sodalite*-ZIF-8 crystals, the powders collected after the centrifugation of the supernatant suspensions for all samples were analyzed by PXRD. In fact, due to the porosity of the substrate and instrumental limitations, only the peaks attributed to the Ti alloy are clearly recognizable on the diffraction patterns collected from the porous scaffolds (Supplementary Figure S2). The XRD patterns obtained from the powders after 24 h are shown in Figure 2; the diffraction peaks at 2θ = 7.3°, 10.4°, 12.7°, 14.7°, 16.4°, and 18.0° associated with the crystal planes (110), (200), (220), (211), (220), (310), and (222) of *sod-*ZIF-8 crystals are clearly recognizable, confirming the presence of a single-phase topology.

**FIGURE 3:**
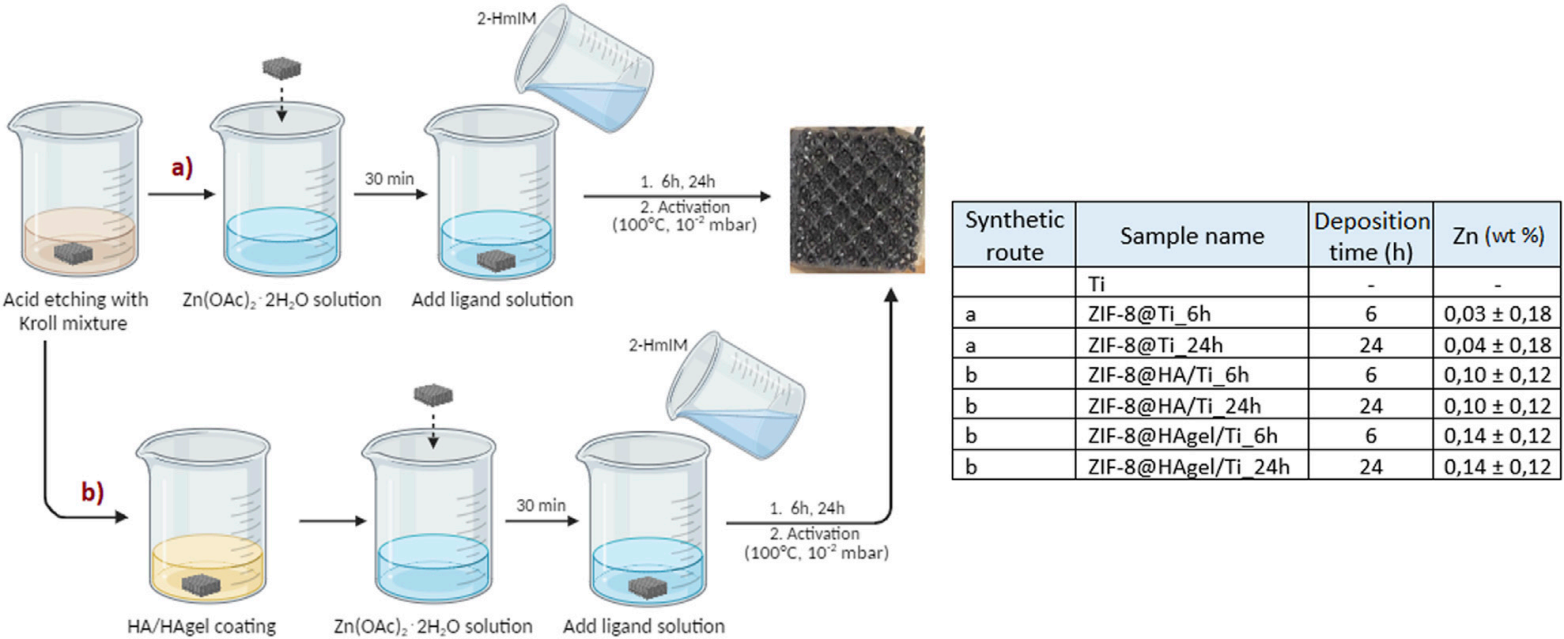
Schematization of the processes used for the preparation of the modified titanium scaffolds. Left: schematization (made with “BioRender”) of the processes used for the preparation of the modified titanium scaffolds; all samples were prepared in triplicate. Right: Zn content determined by MP-AES analyses; the wt% is referred to the weight of the whole scaffold.

Based on these preliminary results, the molar ratio Zn:2-HmIM of 1:16 and deposition times of 6 and 24 h were chosen to evaluate the deposition of ZIF-8 on HA/Ti and HAgel/Ti scaffolds. A scheme of the whole coating procedure with the labels of the obtained products is depicted in Figure 3.

**FIGURE 4:**
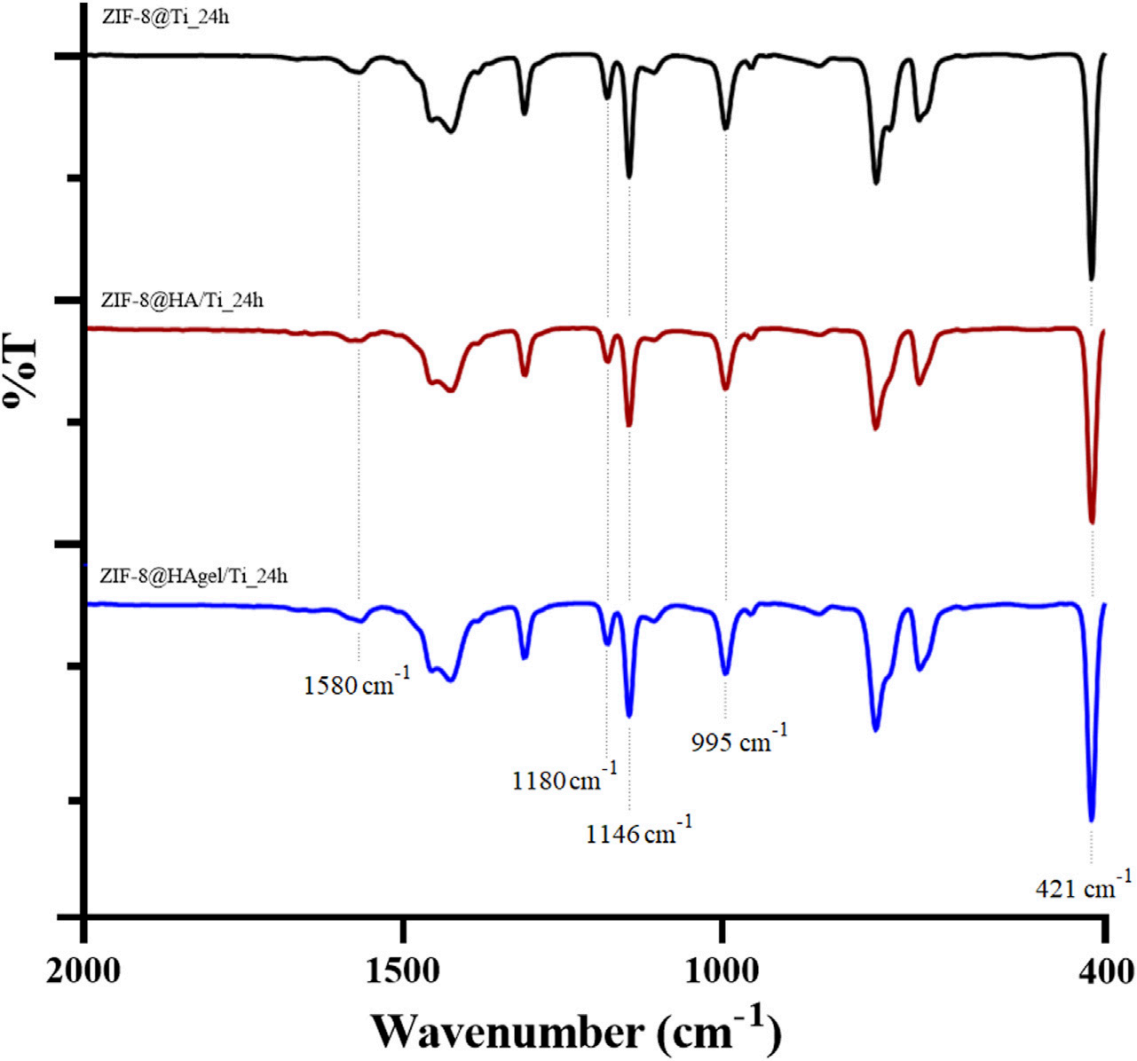
ATR-FTIR spectra of the ZIF-8@Ti_24h (black), ZIF-8@HA/Ti_24h (red), and ZIF-8@HAgel/Ti_24h (blue) powders. The dotted lines represent the most significant bands.

The powders obtained from each synthesis were collected by centrifugation, as described in the experimental part, and characterized by means of infrared spectroscopy and PXRD. The ATR-FTIR spectra of the samples obtained after 24 h are shown in Figure 4, and the spectra of the samples after 6 h are shown in Supplementary Figure S3. The spectra are superimposable and show the characteristic absorption bands associated with the vibrations of the imidazolate units present in *sod-*ZIF-8 except for the Zn-N stretching at 421 cm^−1^ (Astria et al., 2019).

**FIGURE 5:**
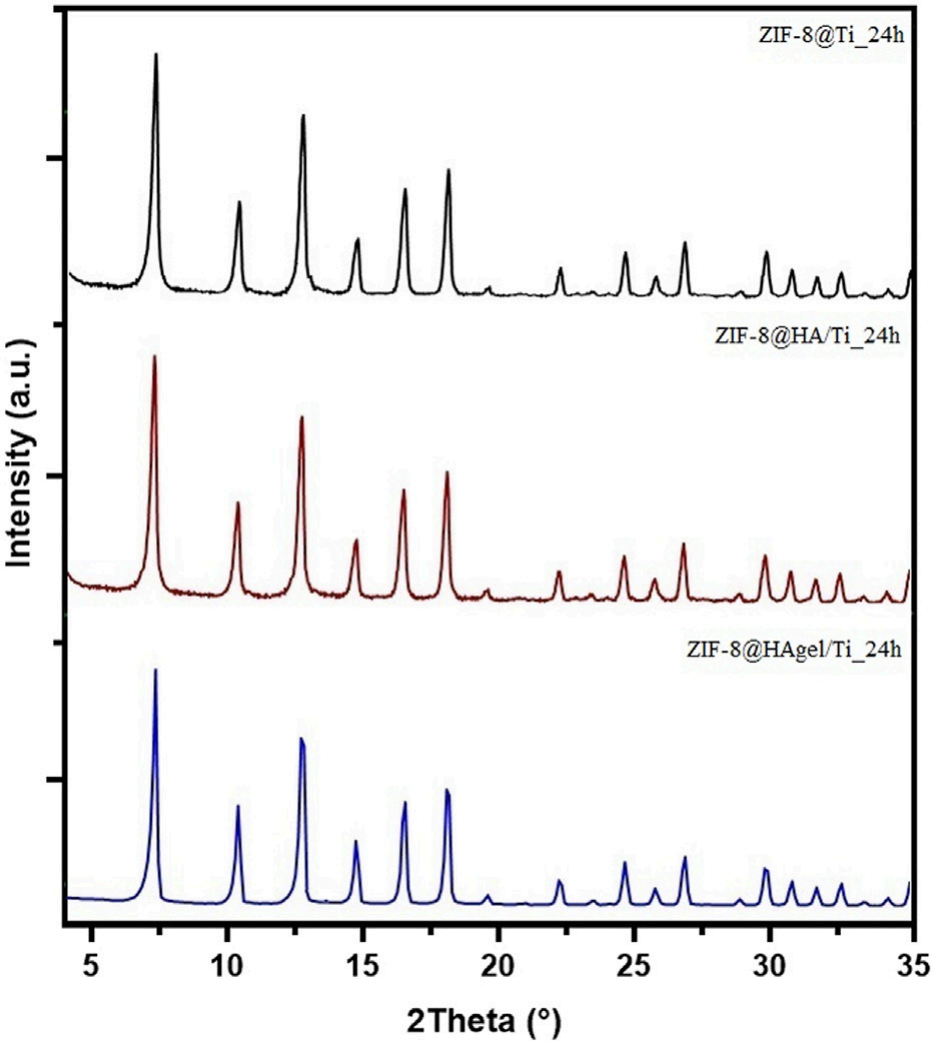
PXRD patterns of the ZIF-8@Ti_24h (black), ZIF-8@HA/Ti_24h (red), and ZIF-8@HAgel/Ti_24h (blue) samples.

The presence of *sod*-ZIF-8 as the only crystalline phase was further confirmed by PXRD analyses. The diffraction patterns of the samples were differentiated based on the contact time, as shown in Supplementary Figure S4 (6 h) and Figure 5 (24 h). The diffraction patterns, for samples collected at the same deposition time, perfectly overlap, highlighting the presence of the same crystalline phase.

**FIGURE 6:**
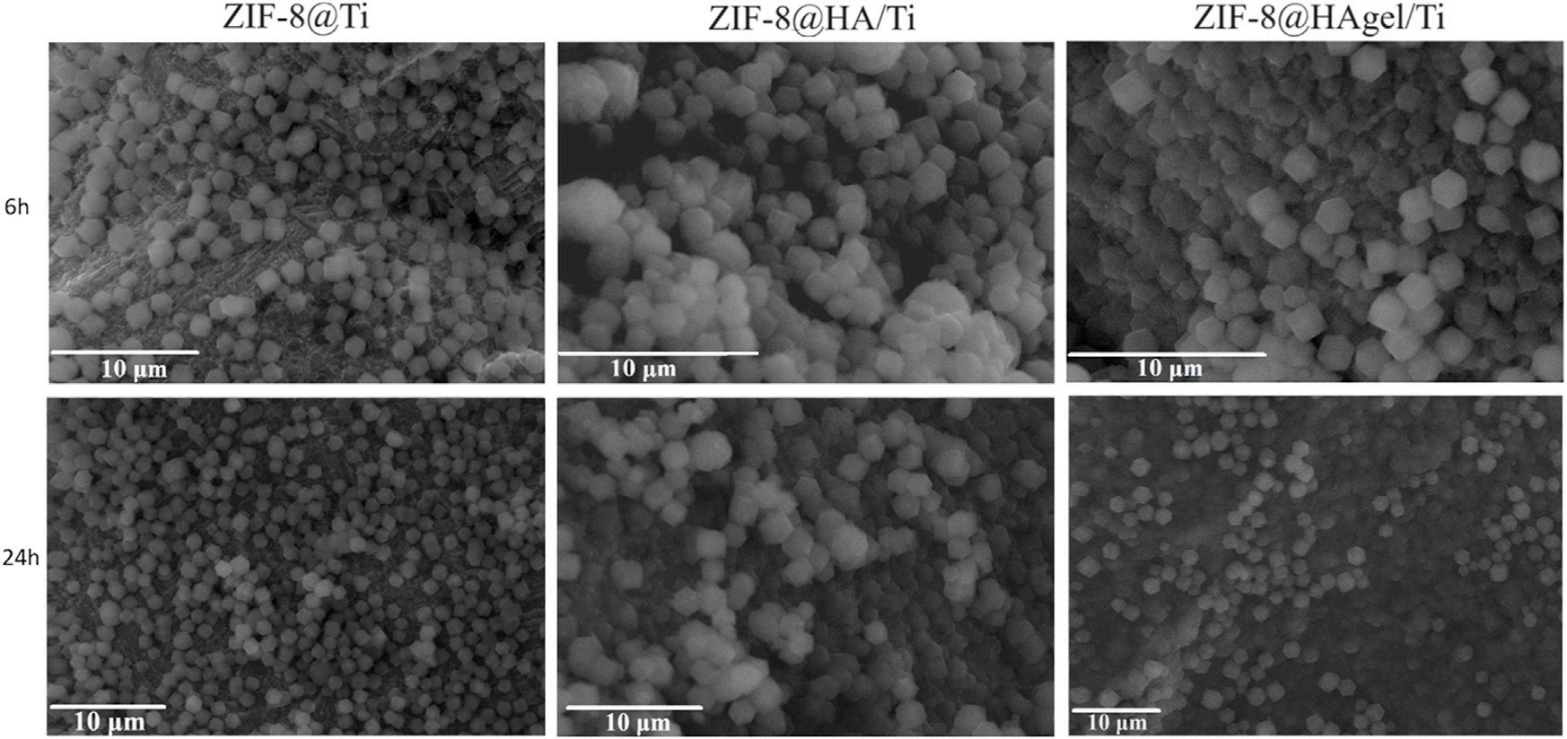
SEM images of different samples after 6 and 24 h contact time (scale bar = 10 µm).

SEM images of the scaffolds (Figure 6) showed the presence of well-dispersed *sod*-ZIF-8 crystals, presenting the characteristic rhombic dodecahedron morphology, on the surface of all the investigated supports and for both the contact times used. The ZIF-8 crystal dimensions range between 1.1 µm of the structures grown directly on Ti scaffolds and 1.4 µm of those grown on coated scaffolds, thus highlighting the role of HA and gelatin in the first stages of nucleation.

The zinc content was evaluated by ICP–AES analysis after treating the samples with nitric acid, as described in the experimental part. To confirm that the coatings have been completely solubilized by the acidic treatment, the processed scaffolds were successively observed by SEM. The image presented in Supplementary Figure S5 revealed only the presence of the Ti substrate. The amount of zinc measured for each sample is reported in Figure 3; both HA and HA/gelatin enhance the adsorption of zinc ions compared to the plain Ti scaffolds. These results are not surprising as the adsorption of Zn ions onto HA is well-documented (Pivarčiová et al., 2015). Furthermore, the amount of zinc adsorbed on each type of sample is similar for both contact times, indicating that the reaction has reached completion after 6 h, which is in line with the work of Pivarciova et al., who reported that Zn (II) adsorption onto HA achieved the maximum value in half an hour. The coating enriched with gelatin showed a higher amount of zinc adsorbed; this result can be explained by the presence of carboxylate groups on the macromolecular chains that further increase ion adsorption.

### 3.2 Cell proliferation

**FIGURE 7:**
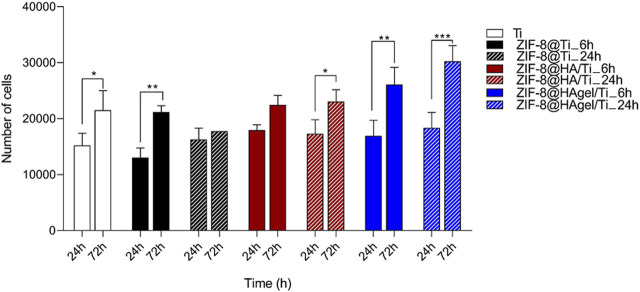
Evaluation of hOB proliferation seeded on materials: i) Ti, ii) ZIF-8@Ti_6h, iii) ZIF-8@Ti_24h, iv) ZIF-8@HA/Ti_6h, v) ZIF-8@HA/Ti_24h, vi) ZIF-8@HAgel/Ti_6h, and vii) ZIF-8@HAgel/Ti_24h, following a time-course of 24 h (T1) and 72 h (T2) (*p < 0.05; **p < 0.005; and ***p < 0.0001; ANOVA test and Tukey’s multiple-comparison test).

The alamarBlue assay allowed to evaluate the cell proliferation of cells seeded on biomaterials i) Ti, ii) ZIF-8@Ti_6h, iii) ZIF-8@Ti_24h, iv) ZIF-8@HA/Ti_6h, v) ZIF-8@HA/Ti_24h, vi) ZIF-8@HAgel/Ti_6h, and vii) ZIF-8@HAgel/Ti_24h. Human primary osteoblast cell proliferation was evaluated at 24 h (T1) and 72 h (T2). No biomaterials showed any cytotoxic effect on hOB proliferation at all time points of analysis, as shown in Figure 7. Titanium (Ti) scaffolds, employed as control, increased cell proliferation by 41% from T1 to T2. The ZIF-8@Ti_6h scaffold seems to stimulate hOB cell proliferation better than ZIF-8@Ti_24h, with a statistical increase in hOB cell proliferation at T2 compared to T1 (62%). Indeed, ZIF-8@Ti_24h did not influence cell proliferation during the time points of analysis.

In ZIF-8@HA/Ti_6h and ZIF-8@HA/Ti_24h, the proliferation of hOBs at T2 increases by 25% and 33%, respectively, compared to T1. ZIF-8@HA/Ti stimulates and maintains increases in hOB proliferation at both times of deposition of ZIF-8 (ZIF-8@HA/Ti_6h and ZIF-8@HA/Ti_24h). Finally, the scaffolds composed of both hydroxyapatite and gelatin, i.e., ZIF-8@HAgel/Ti_6h and ZIF-8@HAgel/Ti_24h, stimulated hOB cell proliferation with a statistical increase by 54% and 64%, respectively, at T2 compared to the first time point of analysis. Proliferation analyses reveal that all the materials are cytocompatible and that ZIF-8@HAgel/Ti materials create a better environment for a significant increase in osteoblast proliferation compared to ZIF-8@HA/Ti and ZIF-8@Ti.

### 3.3 Osteopontin (SPP1) gene expression

Osteopontin, which is the protein encoded by SPP1, represents the major non-collagenous bone protein, promotes cell adhesion and migration, and binds Ca^2+^ (Jang et al., 2009). The expression of the SPP1 gene was evaluated via real-time PCR technology. To this end, hOBs were grown on scaffolds: i) Ti, ii) ZIF-8@Ti_6h, iii) ZIF-8@Ti_24h, iv) ZIF-8@HA/Ti_6h, v) ZIF-8@HA/Ti_24h, vi) ZIF-8@HAgel/Ti_6h, and vii) ZIF-8@HAgel/Ti_24h for 72 h. Titanium scaffold (Ti) was considered a material control group in order to normalize the expression gene data.

**FIGURE 8:**
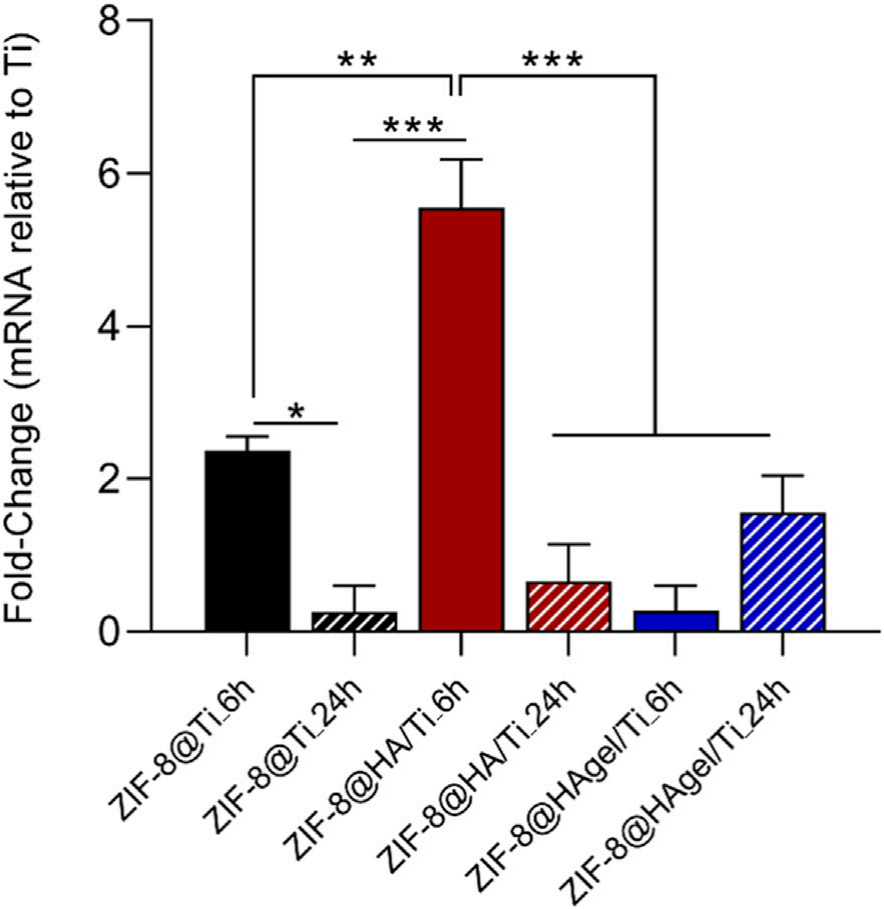
TaqMan real-time PCR was employed to evaluate the expression of the SPP1 gene in hOBs grown on scaffolds: i) Ti, used as control, ii) ZIF-8@Ti_6h, iii) ZIF-8@Ti_24h, iv) ZIF-8@HA/Ti_6h, v) ZIF-8@HA/Ti_24h, vi) ZIF-8@HAgel/Ti_6h, and vii) ZIF-8@HAgel/Ti_24h after 72 h (*p < 0.05; **p < 0.001; and ***p < 0.0001; ANOVA test and Tukey’s multiple-comparison test).

All scaffolds induced the expression of SPP1, compared to the control group represented by hOBs grown on the Ti scaffold (Figure 8), after 72 h. In particular, the SPP1 gene was found to be upregulated in hOBs grown on ZIF-8@Ti_6h, ZIF-8@HA/Ti_6h, and ZIF-8@HAgel/Ti_24h with different fold changes, i.e., FC = 2.36, FC = 5.55, and FC = 1.56, respectively. A statistically significant difference has been observed between hOBs grown on scaffolds composed of ZIF-8@Ti_6h and ZIF-8@Ti_24h, as well as in hOBs grown on ZIF-8@HA/Ti_6h and ZIF-8@HA/Ti_24h. There were no significant differences in SPP1 expression between cells grown on ZIF-8@HAgel/Ti_6h and ZIF-8@HAgel/Ti_24h for 72 h. Overall, hOBs grown on coated-Ti scaffolds were found to express more SPP1 when compared with the uncoated surface, suggesting that coating can provide a good surface for hOB attachment.

### 3.4 Antibacterial assays

Taking into account the results obtained from osteoblast proliferation assay, the antibacterial activity of some selected Ti scaffolds was investigated through different methodologies in order to examine their anti-adhesion potential, bacterial growth dynamics, and anti-biofilm properties against *S. epidermidis*. Assays were carried out with the bare Ti scaffold as a reference control, and the differently functionalized materials coated with ZIF-8 crystals, following a deposition time of 24 h. This contact time was selected because both ZIF-8@HA/Ti_24h and ZIF-8@HAgel/Ti_24h scaffolds proved to significantly stimulate osteoblast proliferation at T2 (72 h) compared to T1 (24 h) in the cellular assays.

**FIGURE 9:**
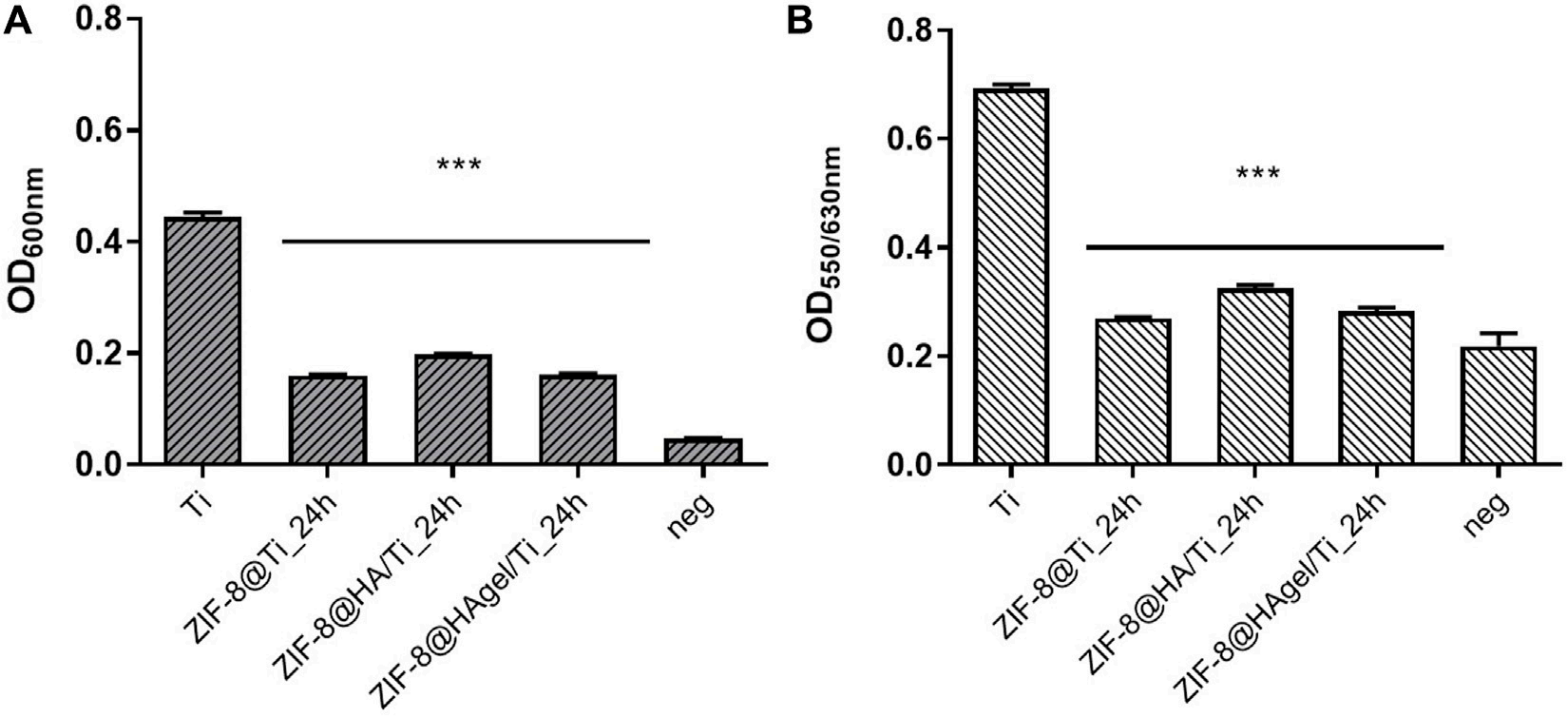
Anti-adhesion assay. Antibacterial performance of the Ti scaffolds incubated for 4 h with a *Staphylococcus epidermidis* culture. Histograms are the OD values at 600 nm, measuring bacterial proliferation in the culture medium **(A)** and at 550/630 nm measuring the metabolic activity of the adhered bacterial cells on the surface of the materials. **(B)** (***p < 0.0001; ANOVA test considered the bare Ti scaffolds as the reference control, and negative samples are aliquots of the culture media).

**FIGURE 10:**
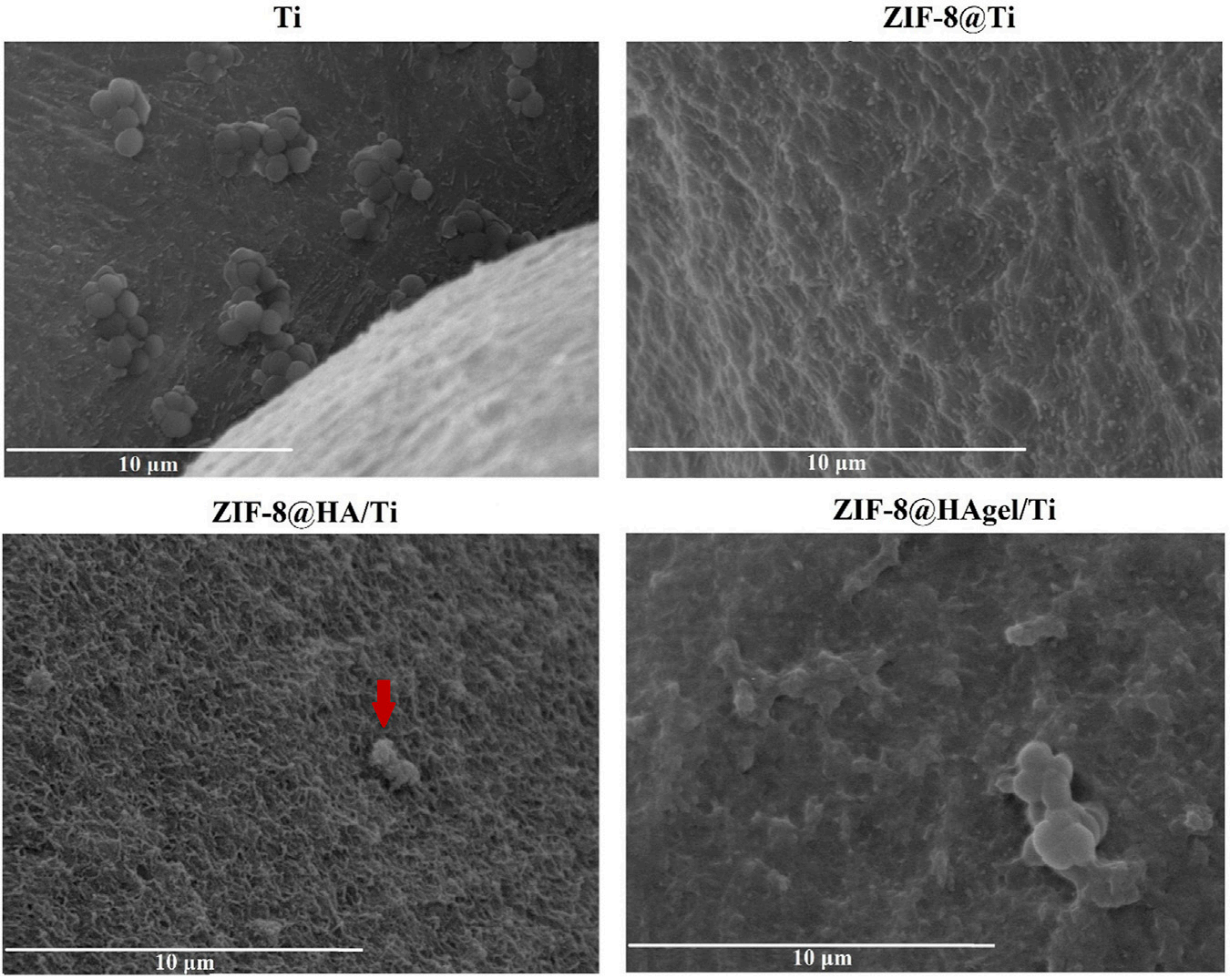
SEM images of *S. epidermidis* attached to the Ti scaffolds. The arrow indicates the corrugated surface of the bacteria onto a layer of HA (a SEM image of the Ti scaffold coated with only HA is reported in Supplementary Figure S6), which is suggestive of the loss of the membrane integrity.

The Ti scaffolds coated with ZIF-8 crystals, regardless of the presence of HA and gelatin A, displayed remarkable antibacterial activity (Figure 9). They significantly reduced the proliferation of *S. epidermidis* in the culture medium with values between 61.7% and 71.1%, indicating that Zn^2+^ ions were effectively released from the ZIF-8-functionalized scaffold, enabling bacterial killing. In addition, ZIF-8-coated scaffolds prevented the adhesion of *S. epidermidis* when compared to the bare Ti scaffold, as demonstrated by the alamarBlue assays. Indeed, only negligible metabolic activity was measured ranging from 10.9% to 22.5%. Following the anti-adhesion analysis, *S. epidermidis* cells attached to the surface of the Ti scaffolds were examined through scanning electron microscopy. SEM images showed differences in cell structures between samples cultured at the different experimental conditions. In detail, bacteria on the bare Ti scaffold were typical cocci, spherical-shaped with a smooth surface, and classically arranged in groupings; the other cells, when observed on the scaffolds, underwent significant deformation, revealing lethal damages (Figure 10). This is a further confirmation that ZIF-8, during the 4 h of incubation in the culture medium, has the ability to release Zn^2+^ ions, which, in turn, trigger a disturbing effect on both bacteria on the scaffold surfaces and those floating on the culture medium, as demonstrated by the anti-adhesion assay. The images also showed the disappearance of the ZIF-8 crystals from the surface after 4 h of incubation in the TBS medium in the anti-adhesion assay. This finding is not surprising as several studies have shown that the decomposition of ZIF-8 occurs in the phosphate-buffered saline solution (pH 7.4), resulting in the formation of insoluble zinc phosphate particles. It is therefore plausible that the same release occurred in the bacterial culture medium having the same pH. The decomposition rate was found to be inversely proportional to the size of the ZIF-8 particles, and for micrometric crystals of 2 μm in size, the most significant changes occur within the first hour of treatment with PBS as around 79% of 2-HmIM is released into the incubation media (Velásquez-Hernández et al., 2019; Butonova et al., 2021; Taheri et al., 2021).

**FIGURE 11:**
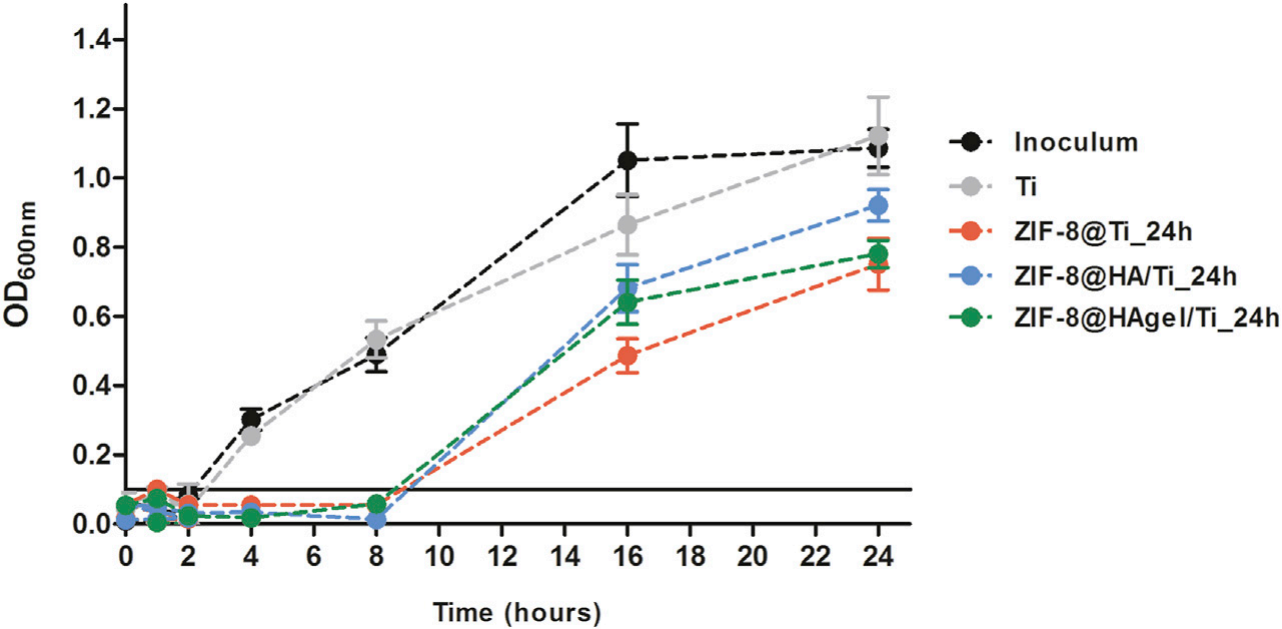
Bacterial growth curves of *S. epidermidis* cultures incubated with the Ti scaffolds and in regular medium as a reference control. A threshold indicating growth above the background level was set at OD_600nm_ = 0.1.

The inhibitory potential of the Zn^2+^ ions released from the ZIF-8-functionalized Ti scaffolds was further investigated by means of a growth kinetic assay on *S. epidermidis* cultures, in which OD_600nm_ measurements were plotted versus different times of incubation such as 1, 2, 4, 8, 16, and 24 h (Figure 11). Cultures exposed to the bare Ti scaffold presented growth kinetics comparable to that of the inoculum control both in terms of duration of the lag phase (2 h) and of OD_600nm_ values at the end of the measurements. On the other hand, cultures incubated with ZIF-8-coated Ti scaffolds displayed a longer lag phase up to 8 h of incubation, and after the resumption of cell growth, bacterial proliferation remained at lower levels compared to control culture. These results are in line with those obtained in the anti-adhesion assays in which the inhibition of cell proliferation was assessed after 4 h of incubation. Notably, the effects of the dissolution of Zn^2+^ ions in the surrounding environment were maintained for a long period of time, enabling for a high antibacterial potential by damaging the bacterial cell wall and the cell membrane and interfering with different intracellular biochemical pathways (Alavijeh et al., 2018; Hoop et al., 2018).

**FIGURE 12:**
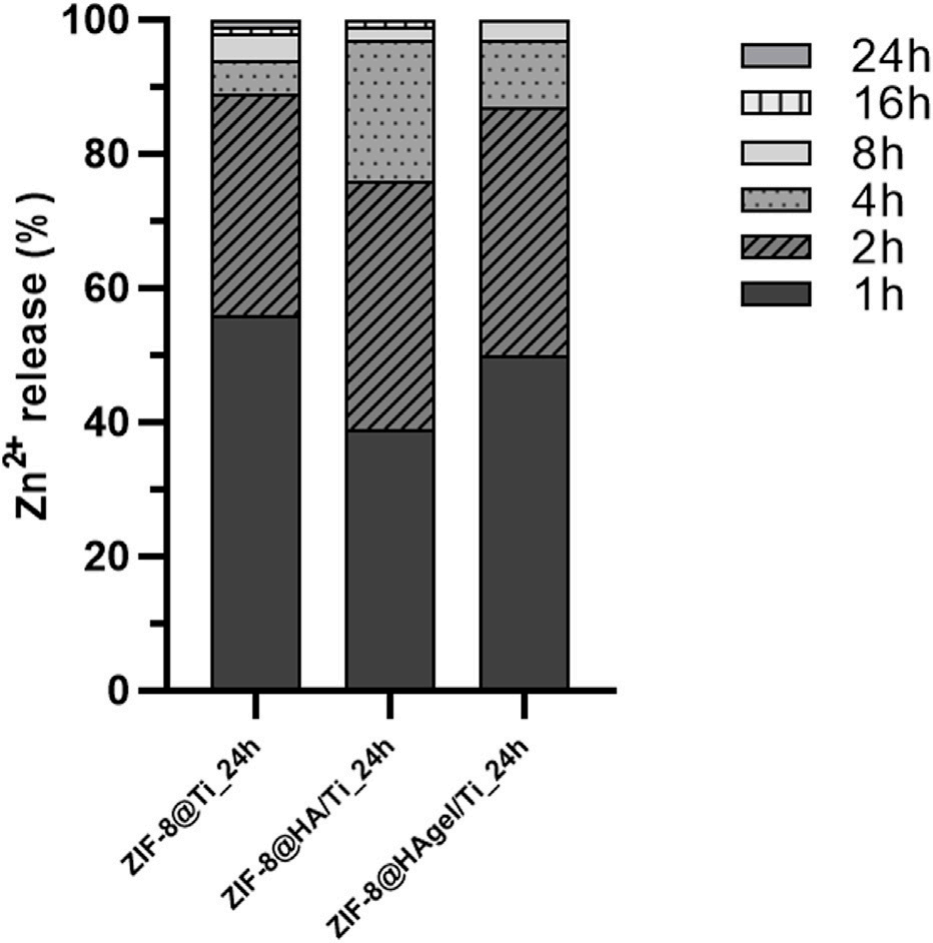
Evaluation of the Zn^2+^ ion-releasing profiles (%) via MP-AES analyses carried out on the harvested solutions of the bacterial growth medium at discrete time points. Histograms are cumulative Zn^2+^ percentages referred to the total amount of released zinc ions.

To support the microbiological evidence of the inhibitory activity of the ZIF-8 functionalized Ti scaffolds, in terms of both adhesion to the materials and delay in the bacterial growth up to 8 h of incubation, the Zn^2+^ ion release kinetics via MP-AES was assessed for the three differently coated scaffolds. Samples were placed in bacterial growth medium at 37°C for a better comparison with the other microbiological studies, and the amount of Zn^2+^ ions released as a function of incubation time was evaluated. Histograms reported in Figure 12 represent the cumulative Zn^2+^ release percentages from each sample and is clearly noticeable that the 97% of zinc is released within the first 8 h of incubation.

**FIGURE 13:**
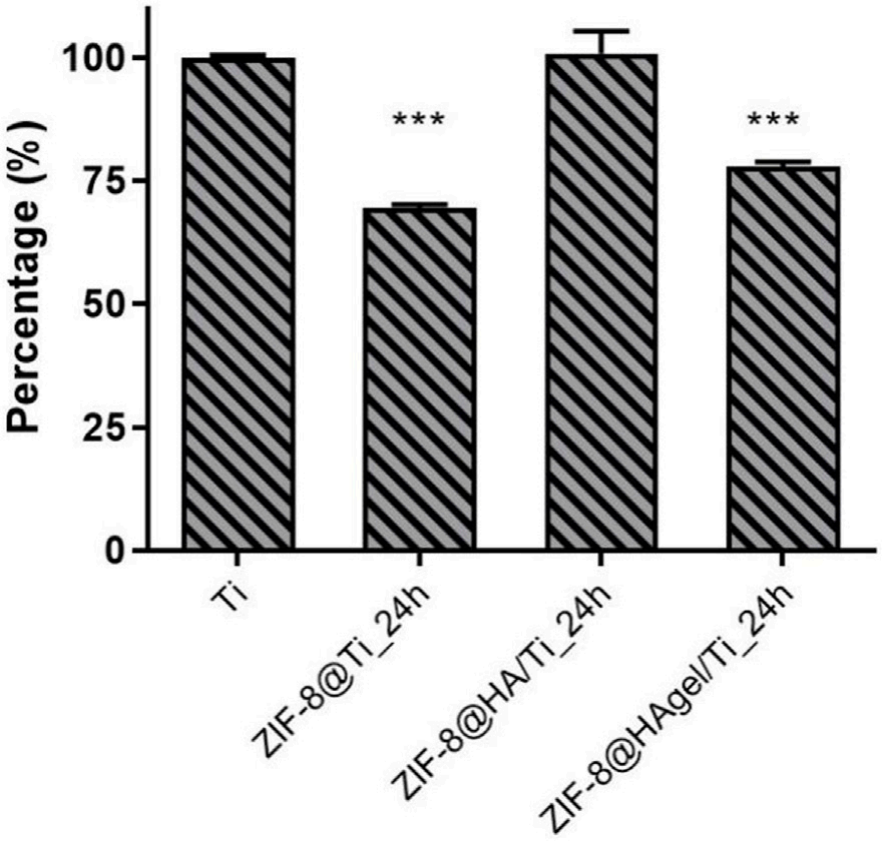
Antibiofilm assay. Values are percentages of the biomass of *S. epidermidis* within the biofilm produced on the tested Ti scaffolds and relative to the plain material. Differences were statistically different for ZIF-8@Ti and ZIF-8@HAgel/Ti (***p < 0.0001; ANOVA test considering the bare Ti scaffolds as the reference control).

Ti scaffolds coated with ZIF-8 crystals were also investigated for their anti-biofilm activity. For this purpose, the Ti scaffolds were incubated with a bacterial suspension for 90 min; thereafter, non-adhered *S. epidermidis* were removed, and the samples were cultured in fresh medium, allowing for biofilm production up to 48 h. The results shown in Figure 13 indicate that the Ti scaffold with ZIF-8 and the scaffold coated with HAgel and ZIF-8 displayed a moderate potential as anti-biofilm materials; the biomass of bacterial cells produced in these experimental conditions was significantly reduced compared to *S. epidermidis* stained on the bare Ti scaffold (range: 22.2%–30.4%, respectively). Differences in anti-biofilm properties between HA- and HAgel-coated scaffolds can be attributed to the greater amount of zinc ions adsorbed on the latter material, as described previously.

## 4 Conclusion

The work here presented demonstrates that porous Ti scaffolds modified with HA and HA/gelatin and coated with ZIF-8 crystals provide a suitable material for bone regeneration, mimicking a physiological environment for osteoblast proliferation. Indeed, all the materials proved to be cytocompatible, according to proliferation analyses; however, an overall improved osteoblast proliferation was measured on scaffolds coated with ZIF-8 crystals, following a deposition time of 24 h compared to 6 h. In addition, among these samples, ZIF-8@HAgel/Ti significantly increased the conditions for osteoblast proliferation compared to ZIF-8@HA/Ti and ZIF-8@Ti. In agreement with the proliferation test, the scaffolds modified with HA and HA/gelatin and coated with ZIF-8 stimulated the expression of the SPP1 gene, an osteogenic marker, compared to the Ti scaffold. In terms of biological performances, scaffolds produced following a deposition time of 6 h allowed for better hOB activity after 72 h.

Moreover, the presence of ZIF-8 on the surface of the differently coated scaffolds, produced following a deposition time of 24 h, allowed for an excellent antibacterial function of the biomaterials. In clinical orthopedics, the bone implant-related infections caused by bacterial adhesion on the surface of the implants are considered a serious issue, and the systemic administration of antibiotics may not guarantee the effective removal of bacteria from the site of the infection. As a consequence, alternative treatments and, above all, preventive strategies enabling for a localized release of the drug are desirable. In this context, ZIF-8-modified Ti scaffolds perfectly meet this requirement as bacterial adhesion to the biomaterials was completely inhibited within the 4-h time frame of the anti-adhesion assay, and bacterial proliferation was completely arrested up to 8 h of incubation as results of the highest release of Zn^2+^ ions in the surrounding environment. This is of clinical relevance because the orthopedic implants are most susceptible to bacterial colonization in this post-surgery time window (Garg et al., 2021).

In conclusion, the herein devised Ti-based biomaterials proved to have a dual effect by promoting osteoconductivity properties in human osteoblast cells and preventing bacterial infections.

## Data Availability

The original contributions presented in this study are included in the article/Supplementary Material, further inquiries can be directed to the corresponding authors.

## References

[B1] Abdelhamid H. N. (2021). Zeolitic imidazolate frameworks (ZIF-8) for biomedical applications: a review. Curr. Med. Chem. 28, 7023–7075. 10.2174/0929867328666210608143703 34102965

[B2] Abdulkareem E. H. Memarzadeh K. Allaker R. P. Huang J. Pratten J. Spratt D. (2015). Anti-biofilm activity of zinc oxide and hydroxyapatite nanoparticles as dental implant coating materials. J. Dent. 43, 1462–1469. 10.1016/j.jdent.2015.10.010 26497232

[B3] Akay S. Yaghmur A. (2024). Recent advances in antibacterial coatings to combat orthopedic implant-associated infections. Molecules 29, 1172. 10.3390/molecules29051172 38474684 PMC10935003

[B4] Alavijeh R. K. Beheshti S. Akhbari K. Morsali A. (2018). Investigation of reasons for metal–organic framework’s antibacterial activities. Polyhedron 156, 257–278. 10.1016/j.poly.2018.09.028

[B5] Astria E. Thonhofer M. Ricco R. Liang W. Chemelli A. Tarzia A. (2019). Carbohydrates@MOFs. Mater Horiz. 6, 969–977. 10.1039/c8mh01611a

[B6] Bigi A. Boanini E. Bracci B. Facchini A. Panzavolta S. Segatti F. (2005). Nanocrystalline hydroxyapatite coatings on titanium: a new fast biomimetic method. Biomaterials 26, 4085–4089. 10.1016/j.biomaterials.2004.10.034 15664635

[B7] Bracci B. (2012). Gelatin-modified biomimetic apatite coatings. J. Biomater. Nanobiotechnol 03, 154–162. 10.4236/jbnb.2012.32021

[B8] Butonova S. A. Ikonnikova E. V. Sharsheeva A. Chernyshov I. Kuchur O. A. Mukhin I. S. (2021). Degradation kinetic study of ZIF-8 microcrystals with and without the presence of lactic acid. RSC Adv. 11, 39169–39176. 10.1039/D1RA07089D 35492461 PMC9044455

[B9] Chen J. Zhang X. Huang C. Cai H. Hu S. Wan Q. (2017). Osteogenic activity and antibacterial effect of porous titanium modified with metal-organic framework films. J. Biomed. Mater Res. A 105, 834–846. 10.1002/jbm.a.35960 27885785

[B10] Chouirfa H. Bouloussa H. Migonney V. Falentin-Daudré C. (2019). Review of titanium surface modification techniques and coatings for antibacterial applications. Acta Biomater. 83, 37–54. 10.1016/j.actbio.2018.10.036 30541702

[B11] Di Matteo V. Di Filippo M. F. Ballarin B. Gentilomi G. A. Bonvicini F. Panzavolta S. (2023). Cellulose/Zeolitic imidazolate framework (ZIF-8) composites with antibacterial properties for the management of wound infections. J. Funct. Biomater. 14, 472. 10.3390/jfb14090472 37754886 PMC10532010

[B12] Furukawa H. Cordova K. E. O’Keeffe M. Yaghi O. M. (2013). The chemistry and applications of metal-organic frameworks. Sci. (1979) 341 341, 1230444. 10.1126/science.1230444 23990564

[B13] Garg D. Matai I. Sachdev A. (2021). Toward designing of anti-infective hydrogels for orthopedic implants: from lab to clinic. ACS Biomater. Sci. Eng. 7, 1933–1961. 10.1021/acsbiomaterials.0c01408 33826312

[B14] Geuli O. Lewinstein I. Mandler D. (2019). Composition-tailoring of ZnO-hydroxyapatite nanocomposite as bioactive and antibacterial coating. ACS Appl. Nano Mater 2, 2946–2957. 10.1021/acsanm.9b00369

[B15] Gomez-Guillen M. C. Gimenez B. Lopez-Caballero M. E. Montero M. P. (2011). Functional and bioactive properties of collagen and gelatin from alternative sources: a review. Food Hydrocoll. 25, 1813–1827. 10.1016/j.foodhyd.2011.02.007

[B16] Han X. Ma J. Tian A. Wang Y. Li Y. Dong B. (2023). Surface modification techniques of titanium and titanium alloys for biomedical orthopaedics applications: a review. Colloids Surf. B Biointerfaces 227, 113339. 10.1016/j.colsurfb.2023.113339 37182380

[B17] Hoop M. Walde C. F. Riccò R. Mushtaq F. Terzopoulou A. Chen X. Z. (2018). Biocompatibility characteristics of the metal organic framework ZIF-8 for therapeutical applications. Appl. Mater Today 11, 13–21. 10.1016/j.apmt.2017.12.014

[B18] Jang J.-H. Castano O. Kim H.-W. (2009). Electrospun materials as potential platforms for bone tissue engineering. Adv. Drug Deliv. Rev. 61, 1065–1083. 10.1016/j.addr.2009.07.008 19646493

[B19] Jian M. Liu B. Liu R. Qu J. Wang H. Zhang X. (2015). Water-based synthesis of zeolitic imidazolate framework-8 with high morphology level at room temperature. RSC Adv. 5, 48433–48441. 10.1039/c5ra04033g

[B20] Kaskel S. (2016). The chemistry of metal–organic frameworks: synthesis, characterization, and applications. Editor Kaskel S. (Weinheim, Germany: Wiley VCH).

[B21] Kaur M. Singh K. (2019). Review on titanium and titanium based alloys as biomaterials for orthopaedic applications. Mater. Sci. Eng. C 102, 844–862. 10.1016/j.msec.2019.04.064 31147056

[B22] Kavanagh N. Ryan E. J. Widaa A. Sexton G. Fennell J. O’Rourke S. (2018). Staphylococcal osteomyelitis: disease progression, treatment challenges, and future directions. Clin. Microbiol. Rev. 31 31, e00084. 10.1128/CMR.00084-17 Staphylococcal osteomyelitis PMC5967688 29444953

[B23] Kheirmand-Parizi M. Doll-Nikutta K. Gaikwad A. Denis H. Stiesch M. (2024). Effectiveness of strontium/silver-based titanium surface coatings in improving antibacterial and osteogenic implant characteristics: a systematic review of *in-vitro* studies. Front. Bioeng. Biotechnol. 12, 1346426. 10.3389/fbioe.2024.1346426 38486866 PMC10937591

[B24] Kouser S. Hezam A. Khadri M. J. N. Khanum S. A. (2022). A review on zeolite imidazole frameworks: synthesis, properties, and applications. J. Porous Mater. 29, 663–681. 10.1007/s10934-021-01184-z

[B25] LeGeros R. Z. (2002). Properties of osteoconductive biomaterials: calcium phosphates. Clin. Orthop. Relat. Res. 395, 81–98. 10.1097/00003086-200202000-00009 11937868

[B26] Li M. Wei Y. Ma B. Hu Y. Li D. Cui X. (2022). Synthesis and antibacterial properties of ZIF-8/Ag-modified titanium alloy. J. Bionic Eng. 19, 507–515. 10.1007/s42235-021-00135-3

[B27] Lien S. M. Ko L. Y. Huang T. J. (2009). Effect of pore size on ECM secretion and cell growth in gelatin scaffold for articular cartilage tissue engineering. Acta Biomater. 5, 670–679. 10.1016/j.actbio.2008.09.020 18951858

[B28] Martini F. Pellati A. Mazzoni E. Salati S. Caruso G. Contartese D. (2020). Bone morphogenetic protein-2 signaling in the osteogenic differentiation of human bone marrow mesenchymal stem cells induced by pulsed electromagnetic fields. Int. J. Mol. Sci. 21, 2104. 10.3390/ijms21062104 32204349 PMC7139765

[B29] Mazzoni E. D’Agostino A. Manfrini M. Maniero S. Puozzo A. Bassi E. (2017). Human adipose stem cells induced to osteogenic differentiation by an innovative collagen/hydroxylapatite hybrid scaffold. FASEB J. 31, 4555–4565. 10.1096/fj.201601384R 28659417

[B30] Mazzoni E. Mazziotta C. Iaquinta M. R. Lanzillotti C. Fortini F. D’Agostino A. (2021). Enhanced osteogenic differentiation of human bone marrow-derived mesenchymal stem cells by a hybrid hydroxylapatite/collagen scaffold. Front. Cell. Dev. Biol. 8 8, 610570. 10.3389/fcell.2020.610570 PMC7849836 33537303

[B31] Mishra P. K. Mishra H. Ekielski A. Talegaonkar S. Vaidya B. (2017). Zinc oxide nanoparticles: a promising nanomaterial for biomedical applications. Drug Discov. Today 22, 1825–1834. 10.1016/j.drudis.2017.08.006 28847758

[B32] Park K. S. Ni Z. Cô A. P. Choi J. Y. Huang R. Uribe-Romo F. J. (2006). Exceptional chemical and thermal stability of zeolitic imidazolate frameworks. Proc. Natl. Acad. Sci. U. S. A. 103, 10186–10191. 10.1073/pnas.0602439103 16798880 PMC1502432

[B33] Phillips J. E. Crane T. P. Noy M. Elliott T. S. J. Grimer R. J. (2006). The incidence of deep prosthetic infections in a specialist orthopaedic hospital. J. Bone Jt. Surg. Br. 88-B 88-B, 943–948. 10.1302/0301-620X.88B7.17150 16799001

[B34] Pivarčiová L. Rosskopfová O. Galamboš M. Rajec P. (2015). Adsorption behavior of Zn(II) ions on synthetic hydroxyapatite. Desalination Water Treat. 55, 1825–1831. 10.1080/19443994.2014.927794

[B35] Ploetz E. Engelke H. Lächelt U. Wuttke S. (2020). The chemistry of reticular framework nanoparticles: MOF, ZIF, and COF materials. Adv. Funct. Mater 30, 1909062. 10.1002/adfm.201909062

[B36] Pourmadadi M. Ostovar S. Eshaghi M. M. Rajabzadeh-Khosroshahi M. Safakhah S. Ghotekar S. (2023). Nanoscale metallic-organic frameworks as an advanced tool for medical applications: challenges and recent progress. Appl. Organomet. Chem. 37, e6982. 10.1002/aoc.6982

[B37] Raja F. N. S. Worthington T. Martin R. A. (2023). The antimicrobial efficacy of copper, cobalt, zinc and silver nanoparticles: alone and in combination. *Biomedical Materials (Bristol)* 18, 045003. Biomed. Mat. 18, 045003. 10.1088/1748-605X/acd03f 37158047

[B38] Shimabukuro M. (2020). Antibacterial property and biocompatibility of silver, copper, and zinc in titanium dioxide layers incorporated by one-step micro-arc oxidation: a review. Antibiotics 9, 716. 10.3390/antibiotics9100716 33092058 PMC7589568

[B39] Sun Y. Zheng L. Yang Y. Qian X. Fu T. Li X. (2020). Metal–organic framework nanocarriers for drug delivery in biomedical applications. Nanomicro Lett. 12, 103. 10.1007/s40820-020-00423-3 34138099 PMC7770922

[B40] Taheri M. Ashok D. Sen T. Enge T. G. Verma N. K. Tricoli A. (2021). Stability of ZIF-8 nanopowders in bacterial culture media and its implication for antibacterial properties. Chem. Eng. J. 413, 127511. 10.1016/j.cej.2020.127511

[B41] Tang H. Yu Y. Zhan X. Chai Y. Zheng Y. Liu Y. (2024). Zeolite imidazolate framework-8 in bone regeneration: a systematic review. J. Control. Release 365, 558–582. 10.1016/j.jconrel.2023.11.049 38042375

[B42] Tao B. Zhao W. Lin C. Yuan Z. He Y. Lu L. (2020). Surface modification of titanium implants by ZIF-8@Levo/LBL coating for inhibition of bacterial-associated infection and enhancement of *in vivo* osseointegration. Chem. Eng. J. 390, 124621. 10.1016/j.cej.2020.124621

[B43] ter Boo G. J. A. Grijpma D. W. Moriarty T. F. Richards R. G. Eglin D. (2015). Antimicrobial delivery systems for local infection prophylaxis in orthopedic- and trauma surgery. Biomaterials 52, 113–125. 10.1016/j.biomaterials.2015.02.020 25818418

[B44] Velásquez-Hernández M. D. J. Ricco R. Carraro F. Limpoco F. T. Linares-Moreau M. Leitner E. (2019). Degradation of ZIF-8 in phosphate buffered saline media. CrystEngComm 21, 4538–4544. 10.1039/c9ce00757a

[B45] Venna S. R. Jasinski J. B. Carreon M. A. (2010). Structural evolution of zeolitic imidazolate framework-8. J. Am. Chem. Soc. 132, 18030–18033. 10.1021/ja109268m 21133378

[B46] Wang A. Walden M. Ettlinger R. Kiessling F. Gassensmith J. J. Lammers T. (2023). Biomedical metal–organic framework materials: perspectives and challenges. Adv. Funct. Mater, 2308589. 10.1002/adfm.202308589 PMC7617264 39726715

[B47] Wang Q. Sun Y. Li S. Zhang P. Yao Q. (2020). Synthesis and modification of ZIF-8 and its application in drug delivery and tumor therapy. RSC Adv. 10, 37600–37620. 10.1039/d0ra07950b 35515141 PMC9057214

[B48] Yang J. Yang Y. (2020). Metal–organic frameworks for biomedical applications. Small 16, 1906846. 10.1002/smll.201906846 32026590

[B49] Zhang X. Chen J. Pei X. Wang J. Wan Q. Jiang S. (2017). Enhanced osseointegration of porous titanium modified with zeolitic imidazolate framework-8. ACS Appl. Mater Interfaces 9, 25171–25183. 10.1021/acsami.7b07800 28696091

