# Supplementary material for: Porous titanium scaffolds modified with Zeolitic Imidazolate Framework (ZIF-8) with enhanced osteogenic activity for the prevention of implant-associated infections
Source: Front Chem. 2024 Aug 29;12:1452670. doi: 10.3389/fchem.2024.1452670 (PMC11390653; doi:10.3389/fchem.2024.1452670)
Supplement: Supplementary file 1 [file DataSheet1.docx]

**Porous titanium scaffolds modified with Zeolitic Imidazolate Framework (ZIF-8) with enhanced osteogenic activity for the prevention of implant-associated infections**

Valentina Di Matteo^1^, Maria Francesca Di Filippo^2^, Barbara Ballarin^1^, Francesca Bonvicini^3^, Maria Rosa Iaquinta^4^, Silvia Panzavolta^2^, Elisa Mazzoni^5,6^*, Maria Cristina Cassani^1^*

^1^Department of Industrial Chemistry “Toso Montanari”, University of Bologna, Bologna, Italy,^2^ Department of Chemistry “G. Ciamician”, University of Bologna, Bologna, Italy, ^3^ Department of Pharmacy and Biotechnology, University of Bologna, Bologna, Italy, ^4^ Department of Medical Sciences, Section of Experimental Medicine, University of Ferrara, Ferrara, Italy, ^5^ Department of Chemical, Pharmaceutical and Agricultural Sciences, University of Ferrara, Ferrara, Italy, ^6^ Laboratory for Technologies of Advanced Therapies (LTTA), University of Ferrara, Ferrara, Italy.

*CORRESPONDENCE

Elisa Mazzoni and Maria Cristina Cassani

[elisa.mazzoni@unife.it](mailto:elisa.mazzoni@unife.it); maria.cassani@unibo.it

Supplementary

**
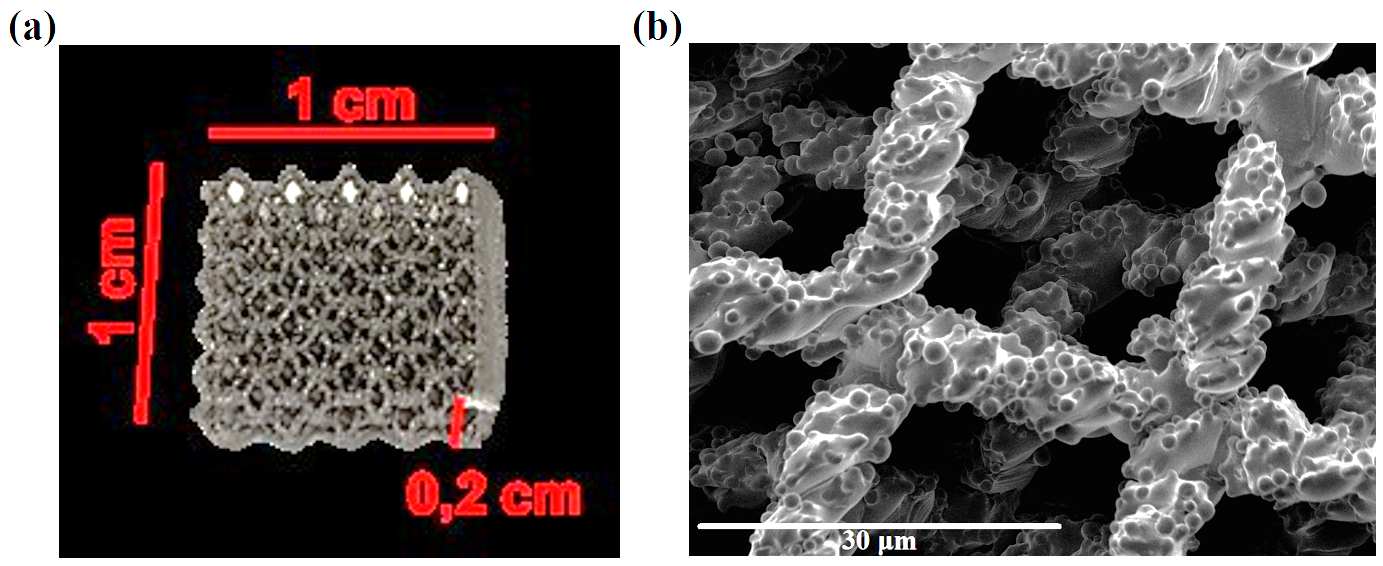
**

**Figure S1**: (a) optical micrograph of the pristine scaffold and its dimensions; (b) SEM image collected on the pristine scaffold.

**
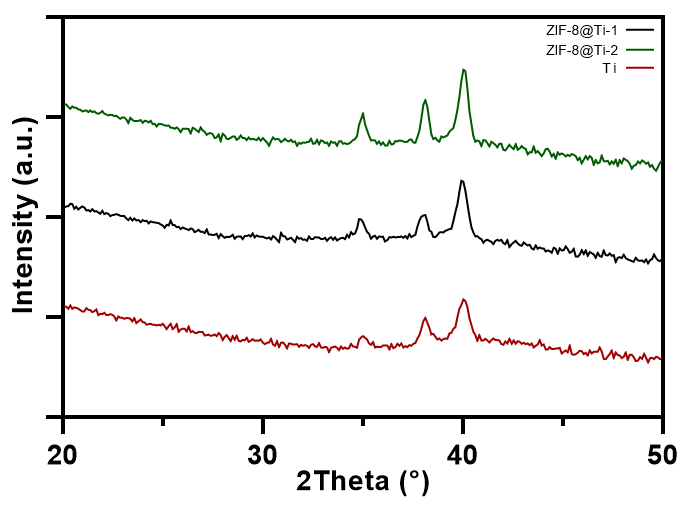
**

**Figure S2**: XRD comparison between pristine Ti scaffold (red), ZIF-8@Ti-1_24h scaffold (black), ZIF-8@Ti-2_24h scaffold (green).

**
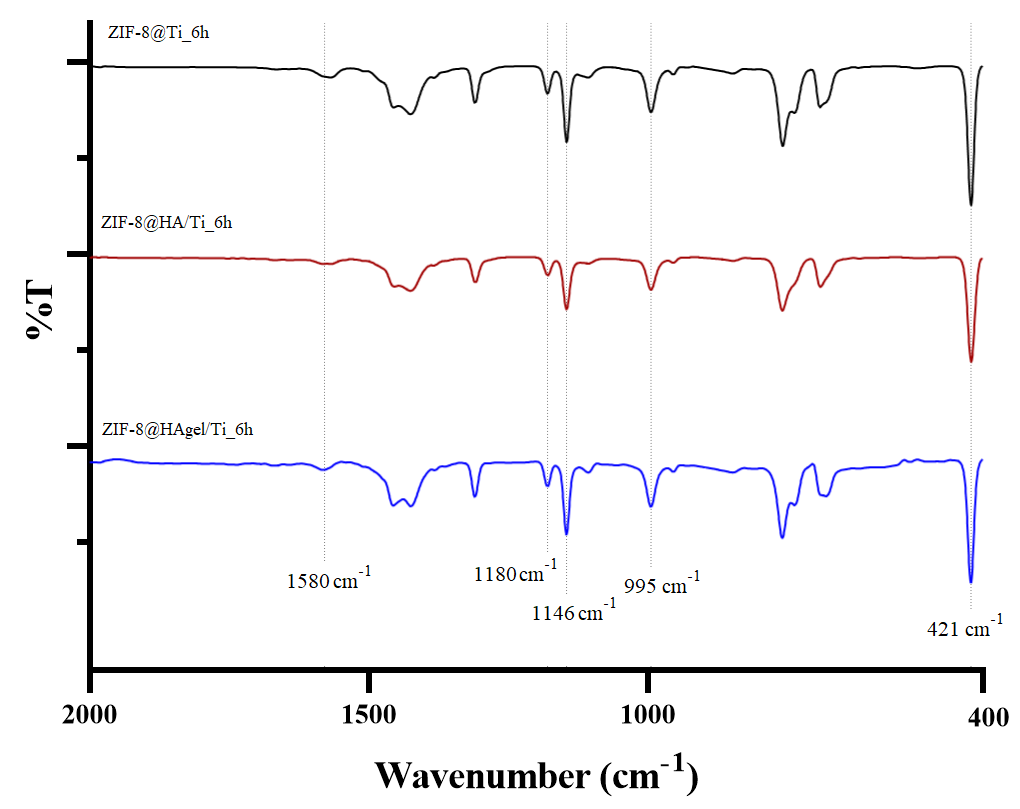
**

**Figure S3**: ATR-FTIR comparison between ZIF-8@Ti_6h (black), ZIF-8@HA/Ti_6h (red), ZIF-8@HAgel/Ti_6h (blue). The dotted lines represent the most significant bands.


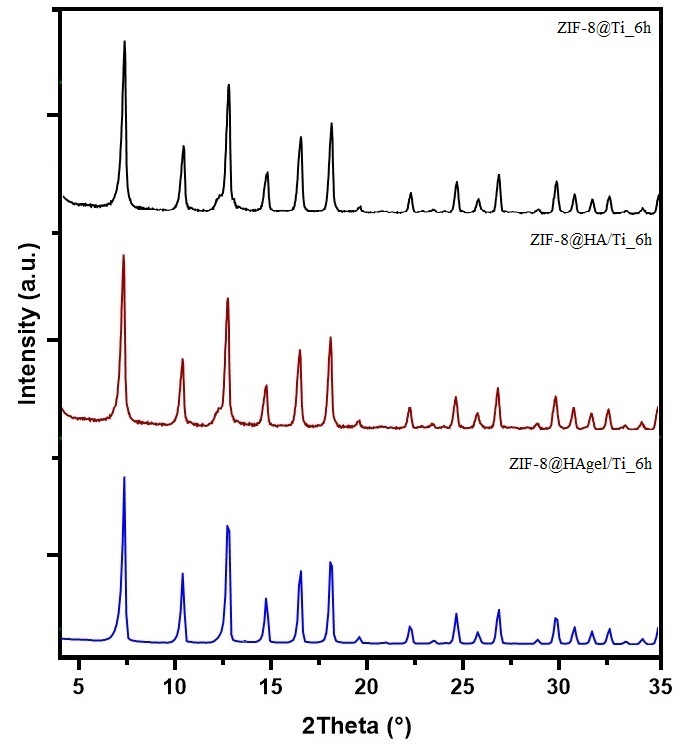


**Figure S4**: PXRD comparison between ZIF-8@Ti_6h (black), ZIF-8@HA/Ti_6h (red), ZIF-8@HAgel/Ti_6h (blue).


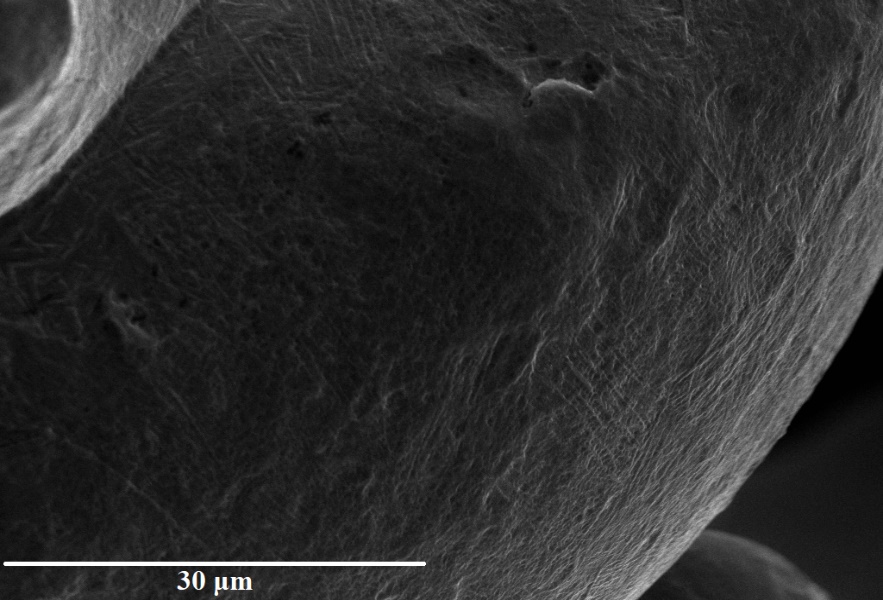


**Figure S5:** SEM image of the Ti scaffold after treatment with 1.0 M nitric acid.


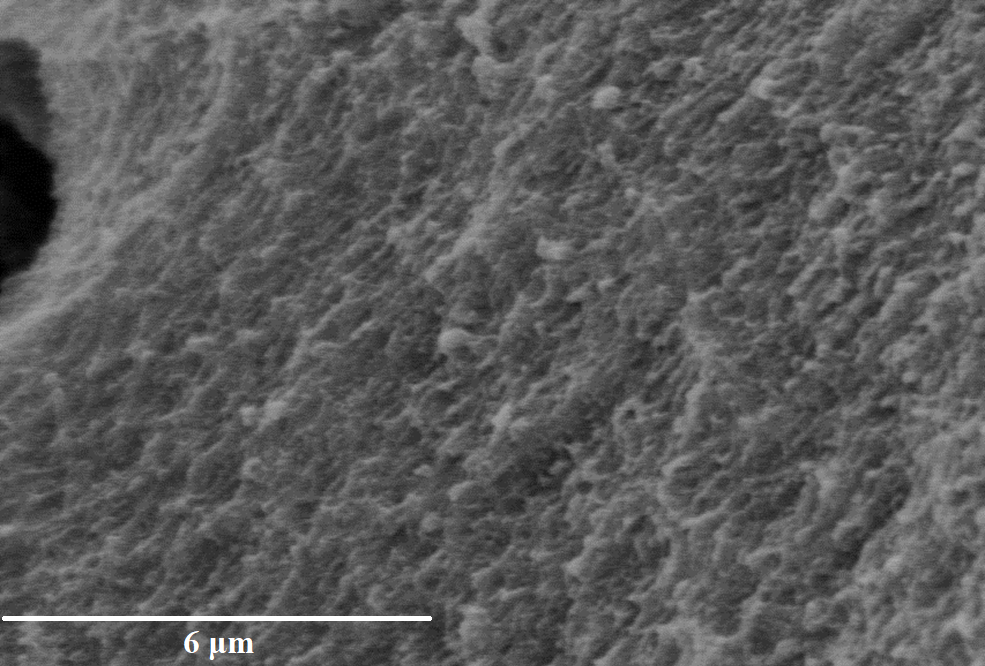


**Figure S6**: SEM image of a Ti scaffold coated with only HA.
